# Forebrain AR Deletion Restores PR Expression but not Reproduction in Prenatally Androgenized Female Mice

**DOI:** 10.1210/endocr/bqaf161

**Published:** 2025-11-05

**Authors:** Emily E A Lott, Melanie Prescott, Kyoko Potapov, David J Handelsman, Kelly A Glendining, Rebecca E Campbell

**Affiliations:** Centre for Neuroendocrinology and Department of Physiology, School of Biomedical Sciences, University of Otago, Dunedin 9054, New Zealand; Centre for Neuroendocrinology and Department of Physiology, School of Biomedical Sciences, University of Otago, Dunedin 9054, New Zealand; Centre for Neuroendocrinology and Department of Physiology, School of Biomedical Sciences, University of Otago, Dunedin 9054, New Zealand; Andrology Department, ANZAC Research Institute, University of Sydney, Concord Hospital, New South Wales 2139, Australia; Centre for Neuroendocrinology and Department of Physiology, School of Biomedical Sciences, University of Otago, Dunedin 9054, New Zealand; Centre for Neuroendocrinology and Department of Physiology, School of Biomedical Sciences, University of Otago, Dunedin 9054, New Zealand

**Keywords:** PCOS, prenatal androgen, androgen receptor, progesterone receptor, reproduction

## Abstract

Prenatal androgen excess (PNA), an etiologic factor for polycystic ovary syndrome (PCOS), is implicated in programming long-term reproductive deficits in females such as anovulation, subfertility, and hyperandrogenism. Impaired steroid hormone feedback is a key neuroendocrine feature suspected to underpin the development of reproductive dysfunction in both clinical PCOS and in PNA mice exposed to dihydrotestosterone during late gestation. PNA is suspected to act in the brain to program the impaired sensitivity of the GnRH neuronal network to progesterone negative feedback, centrally dysregulating the hypothalamic-pituitary-ovarian axis controlling reproduction. To test the hypothesis that androgen-sensitive neurons mediate PNA programming, we generated PNA female mice with a neuron-specific deletion of androgen receptors (AR) (NeurARKO) using Cre-lox transgenics. Following confirmation of embryonic AR deletion, PNA NeurARKO females were reproductively phenotyped and assessed for changes in progesterone receptor expression in the brain. PNA-induced reproductive traits including delayed pubertal onset, acyclicity, altered ovarian morphology, and subfertility were not different between NeurARKO and wild-type mice. In contrast, downregulation of progesterone receptor expression in PNA wild-type mice was protected against in PNA NeurARKO mice. Together, these findings suggest that although neuronal AR may contribute to PCOS-like impaired sensitivity to progesterone feedback, their deletion alone is insufficient to rescue reproductive dysfunction associated with PCOS.

## Introduction

Polycystic ovary syndrome (PCOS) affects an estimated 20% of reproductive-aged women and is characterized by hyperandrogenism, oligo- or anovulation, and polycystic ovarian morphology (1, 2). In addition to metabolic and psychological comorbidities, women with PCOS commonly exhibit increased LH pulse frequency (3), reflecting elevated GnRH neuron activity. GnRH neurons within the hypothalamus govern the hypothalamic-pituitary-ovarian (HPO) axis, a neuroendocrine signaling cascade that coordinates female reproduction. In patients with hyperandrogenic PCOS, higher concentrations of ovarian steroid hormones are required to slow LH pulses compared to those without PCOS (4), indicating fundamental dysregulation of the HPO axis. It is hypothesized that hyperandrogenism impairs the sensitivity of the steroid-sensitive GnRH neuronal network to gonadal hormone feedback, leading to elevated GnRH/LH activity, which in turn disrupts ovarian function (5).

Although the etiology is unclear, daughters born to women with PCOS have a 5-fold increased risk of developing PCOS themselves (6), implicating exposure to androgen excess during the prenatal period as an etiologic factor. Moreover, prenatal androgen excess (PNA) using the nonaromatizable androgen DHT programs long-term reproductive dysfunction in females across several mammalian species (7). In female mice, PNA also programs neuroendocrine features including elevated LH pulse frequency (8), impaired sensitivity to gonadal hormone feedback (8-10), and pathological wiring of the GnRH neuronal network (8, 11-13). PCOS-like reproductive and metabolic features also develop in adult female mice chronically exposed to DHT from the peripubertal period (referred to as peripubertally androgenized [PPA]), supporting the contribution of hyperandrogenism to reproductive dysfunction. Interestingly, PPA female mice exhibit severe metabolic features and impaired cyclicity, but an absence of elevated GnRH/LH pulse frequency (14), indicating that the critical window and duration of hyperandrogenism exposure programs differential PCOS-like traits.

Blockade of androgen signaling ameliorates reproductive features of PCOS, establishing androgen receptors (AR) as a key therapeutic target. Restoration of reproductive cycling and the sensitivity of the GnRH neuronal network to steroid hormone feedback has been shown in PNA female mice following long-term pharmacological antagonism of AR (15-17). In corroboration, global AR knockout (ARKO) mice are protected from developing PCOS-like traits in both the PNA (18) and PPA (19) models of PCOS. However, the specific androgen-sensitive targets mediating the pathogenic programming of androgen excess is unclear.

Female PPA mice with a neuron-specific knockout of AR (NeurARKO) exhibit amelioration of androgen excess-induced reproductive and metabolic dysfunction, highlighting the brain as a key mediator of androgen excess. Caldwell et al (2017) generated NeurARKO mice by crossing AR-floxed (AR^fl/fl^) mice with CamKllα-Cre mice and reported a restoration of corpora lutea (CL) and metabolic function. Additionally, NeurARKO mice generated using Synapsin1-Cre and exposed to chronic DHT from 2 months of age exhibited partial restoration of glucose intolerance (20), but reproductive dysfunction persisted (21).

Evidence implicates the brain in mediating androgen excess in other mouse models of PCOS; however, its role in the PNA model remains unclear. It is hypothesized that PNA drives impaired sensitivity to progesterone negative feedback in GnRH neuron afferents, subsequently driving GnRH/LH hyperactivity (5, 22). Reduced mRNA and protein expression of progesterone receptors (PR) and an increase in AR have been reported in the rostral periventricular nucleus of the third ventricle (RP3V) and arcuate nucleus (ARC), which contain excitatory GnRH neuron afferents (8, 10, 23, 24). In addition, PNA has been consistently reported to program an increase in GABAergic innervation and neurotransmission to GnRH neurons (8, 11-13, 25-28), a phenotype reversed by pharmacological blockade of AR (11) and by AR deletion in GABAergic neurons (25). Therefore, while the brain is a target of PNA programming, its role in mediating the development of reproductive dysfunction is unclear.

This study aimed to investigate whether androgen signaling in neurons is required to mediate the development of PNA-induced PCOS-like features. We generated NeurARKO offspring by crossing CamKllα-Cre^+/−^ and AR^fl/Y^ mice and validated hypothalamic loss of AR in embryonic and adult mice using immunohistochemistry. We hypothesized that NeurARKO females exposed to PNA would be protected from developing PCOS-like features including delayed pubertal onset, acyclicity, hyperandrogenism, subfertility, altered ovarian morphology, and reduced PR expression in hypothalamic nuclei.

## Materials and Methods

### Experimental Animals

Mice were housed in temperature- (22 ± 1 °C) and humidity-controlled rooms with 12-hour light/dark cycles and ad libitum access to food and water at the University of Otago Biomedical Research Facility, Eccles Building (Dunedin, New Zealand). All experimental protocols and procedures were approved by the University of Otago Animal Ethics Committee (Dunedin, New Zealand) and all experiments were performed in accordance with Australian & New Zealand Council for the Care of Animals in Research and Teaching guidelines and regulations.

### Neuron-specific ARKO Mice

To generate mice with NeurARKO, AR^fl/fl^ mice (29) were mated with transgenic CamKllα-Cre mice (30). Experimental animals were produced by crossing heterozygous CamKllα-Cre^+/−^; AR^fl/wt^ females with hemizygous AR^fl/Y^ males. CamKllα-Cre^−/−^; AR^fl/fl^ females were used as controls (referred to as wild-type [WT]).

CamKllα-Cre-mediated excision of AR was confirmed using NiDAB chromogenic immunohistochemistry in brain sections from embryonic day 16 (E16) WT male (n = 2), WT female (n = 2), NeurARKO male (n = 3), and NeurARKO female mice (n = 3) from 4 litters. In addition, excision of AR was assessed by immunohistochemistry in adult female WT (n = 5) and NeurARKO (n = 4) mice.

### Prenatal Androgen Treatment to Model PCOS

Prenatally androgenized female mice were generated as previously described (31). Briefly, time-mated pregnant dams were subcutaneously injected with 250 µg of DHT (A8380-1G; Sigma-Aldrich) in 100 μL sesame oil to generate PNA mice, or sesame oil alone to generate vehicle (Veh) control female offspring, on gestational days 16, 17, and 18. Experimental groups comprised female offspring from 39 litters and included Veh WT (n = 5-13), Veh NeurARKO (n = 4-8), PNA WT (n = 5-15), and PNA NeurARKO (n = 4-8) mice.

### Assessment of Pubertal Onset and Fertility

Following weaning, reproductive function was assessed in Veh WT (n = 8-13), Veh NeurARKO (n = 6-8), PNA WT (n = 8-15), and PNA NeurARKO (n = 5-8) females. Pubertal timing was assessed by daily examination for the age of vaginal opening. In addition, first estrus was identified by daily vaginal cytology or until postnatal day (PND) 50. Estrous cyclicity was then assessed by daily examination of vaginal cytology at PND60-80 (32). Body weight was recorded every 5 days from PND25-80. The fertility and fecundity of adult Veh WT (n = 5), Veh NeurARKO (n = 4), PNA WT (n = 5), and PNA NeurARKO (n = 4) mice was tested in adulthood by pairing females with untreated stud males for 3 months. The time to first litter, numbers of litters produced, the average number of pups per litter, and total number of pups weaned were recorded over the mating period.

### Steroid Hormones

A terminal blood sample was collected from adult Veh WT (n = 10), Veh NeurARKO (n = 5), PNA WT (n = 9), and PNA NeurARKO (n = 4) female mice in diestrus to assess serum levels of testosterone, androstenedione, progesterone, estrone, and estradiol. The samples were stored frozen until sent to the ANZAC Research Institute (Sydney, Australia) where liquid chromatography-tandem mass-spectrometry (LC-MS) was performed, as previously described (33). The detection limit (DL) for each steroid was: testosterone, 0.01 ng/mL; androstenedione, 0.05 ng/mL; progesterone, 0.05 ng/mL; estrone, 0.5 pg/mL; and estradiol, 0.5 pg/mL. Results below the DL were replaced by DL/√2, as previously validated (34).

### Ovarian Histology

Stereology was used to quantify ovarian structures. Intact ovaries were dissected from perfusion fixed adult Veh WT (n = 8), Veh NeurARKO (n = 7), PNA WT (n = 8), and PNA NeurARKO (n = 6) female mice in diestrus, as determined by vaginal cytology, and postfixed for 1 hour in 4% paraformaldehyde. Five-micron-thick ovarian sections were collected in 50-µm intervals and stained with hematoxylin and eosin (GeminiTM AS Automated Slide Stainer, EprediaTM). Classification of ovarian structures were performed as published (35). Each section was visualized using light microscopy (Olympus BX51) with a 20× objective, and the number of primordial and primary follicles were quantified in all sections. Every section was then imaged with a 4× objective. Using ImageJ software (National Institutes of Health, Bethesda, Maryland), secondary, antral, and atretic follicles were counted when the nucleolus was present in every fourth section, which were 200 µm apart. This method ensured that follicles were only counted once, as previously described (36). Atretic follicles included those with a degenerative oocyte and/or more than 10% pyknotic granulosa cells, as characterized previously (37). The number of preovulatory follicles and CL were counted in every sixth section, which were 300 µm apart. In addition, the percentage of tissue in the 2 largest of 5 middle sections of each ovary made up of corpora lutea was also quantified.

### Brain Collection and Sectioning

To validate developmental AR loss, pregnant dams were perfused on the 16th day of pregnancy with 4% paraformaldehyde (PFA). Embryos were dissected out of the uterine horns on ice, and their brains were further dissected from the skull, postfixed in 4% PFA overnight, and then cryoprotected in a 30% sucrose/Tris-Buffered saline solution for a further 24 hours at 4 °C. E16 brain sections were cut into 2 series of 20-μm-thick sections using a cryostat and sections were immediately thaw mounted onto microscope slides.

In the experimental cohort, PND80 Veh WT (n = 8), Veh NeurARKO (n = 7), PNA WT (n = 7), and PNA NeurARKO (n = 5) female mice underwent transcardial perfusion with 4% PFA. The resulting perfusion fixed brains were dissected from the skull, postfixed in 4% PFA overnight and then cryoprotected. The perfusion fixed brains were then cut into 3 series of 30-μm-thick sections using a freezing microtome.

### Chromogen Immunohistochemistry

Two series of adult brain sections containing the rostral-to-caudal extent of the hypothalamus were used to perform single-label, free-floating immunohistochemistry for AR and PR, as previously reported (8). In addition, the same protocol was employed to label AR in 1 series of E16 brain sections on microscope slides. Primary antibodies, including monoclonal anti-AR (Abcam Cat# ab105225, RRID:AB_2782991) or polyclonal anti-PR (Genscript Cat# RC269, RRID:AB_2924988), were used at a concentration of 1:1000 and 1:2500, respectively. Primary antibody omission served as a negative control. The secondary antibody, biotinylated goat anti-rabbit (Vector Laboratories Cat# BA-1000, RRID:AB_2313606) was used at a concentration of 1:800 for anti-AR and 1:250 for anti-PR. Chromagen labelling was achieved using 1:100 of the A/B Vectastain Elite kit (Vector Laboratories Cat# PK-6100, RRID:AB_2336819) and 3,3′-diaminobenzidine (DAB) enhanced with nickel.

### Microscopy and Image Analysis

Image acquisition of bright field microscopy with a 10× objective was performed using an Olympus BX-51 microscope. In E16 brain sections, qualitative assessment of AR expression was made in captured images from the paraventricular nucleus (PVN), ARC, and paraventricular nucleus of the thalamus (PVT). In adult mice, quantification of steroid hormone receptor-positive nuclei was performed in 2 representative sections from bilateral hemispheres (where possible) from each hypothalamic nucleus analyzed. Images of AR and PR-labelled cells were captured in brain sections of the anteroventral periventricular nucleus (AVPV), rostral periventricular nucleus (rPeN), caudal periventricular nucleus (cPeN), rostral arcuate nucleus (rARC), middle arcuate nucleus (mARC), and caudal arcuate nucleus (cARC) with reference to the Paxinos Mouse Brain Atlas. ImageJ software was used to define the nuclei regions and quantification was automated using QuPath software.

### Statistical Analysis

All statistical analysis and graphing was performed with PRISM software (v9.0 Graphpad software). Data normality was determined by the Shapiro–Wilk test. To assess whether the number of AR-labelled cells was reduced in NeurARKO mice, an unpaired *t*-test was used. To assess the effect of PNA treatment, the NeurARKO genotype, and the PNA × NeurARKO interaction, a 2-way ANOVA test was used. If there was a significant PNA × NeurARKO interaction, Tukey multiple comparisons test was used for post hoc analysis. All data are represented as mean ± SEM. Statistical significance was determined as *P* < .05.

## Results

### Hypothalamic Deletion of AR in Embryonic and Adult NeurARKO Mice

**Figure 1.**
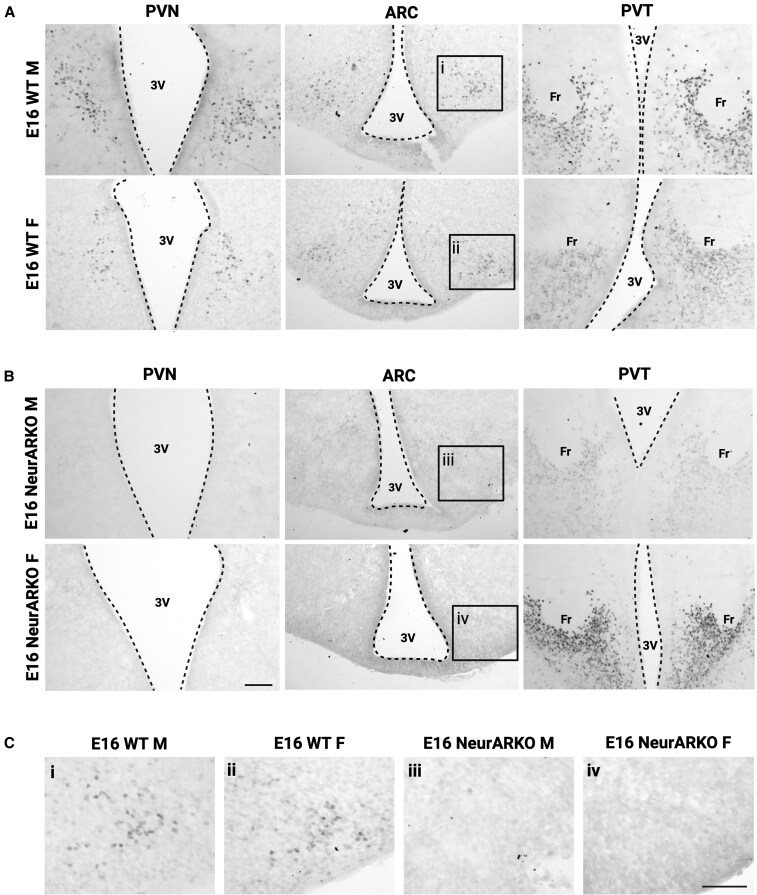
Validation of embryonic androgen receptor knock out in hypothalamic neurons. (A) Representative brightfield images (10× magnification, scale bars = 100 μm) of AR-labelled cells in regions of the forebrain in E16 male and female WT mice. (B) Representative brightfield images (10× magnification, scale bars = 100 μm) of AR-labelled cells in regions of the forebrain in E16 male and female NeurARKO mice. (C) Representative brightfield images (20× magnification, scale bars = 50 μm) of AR-labelled cells within the ARC of male and female WT mice, and loss of AR-labelled cells in NeurARKO mice. Abbreviations: 3V, third ventricle; ARC, arcuate nucleus; Fr, fasciculus retroflexus; PVN, paraventricular nucleus; PVT, paraventricular thalamic nucleus.

NiDAB immunohistochemistry for AR was performed on brain sections from E16 WT and NeurARKO male and female mice to determine the embryonic expression pattern of AR, and whether CamKllα-Cre-mediated AR deletion occurs at this age. In E16 WT mice, AR-labelled cells were consistently observed in the PVN, ARC, and PVT (Fig. 1A and 1C), as well as the septum, dorsal medial hypothalamus, and subthalamic nucleus (data not shown). In all E16 NeurARKO mice, AR-labelled cells were absent from the PVN and ARC (Fig. 1B and 1C), indicating embryonic AR deletion within the hypothalamus. However, AR labelling was observed in the PVT (Fig. 1B), septum and subthalamic nucleus (data not shown) of NeurARKO mice, demonstrating that CamKllα-Cre activity is specific to the forebrain at E16. As the initial insult of DHT exposure in the PNA model occurs at E16, NeurARKO mice are a useful model to investigate the role of hypothalamic AR signaling in mediating the developmental effect of androgen excess.

**Figure 2.**
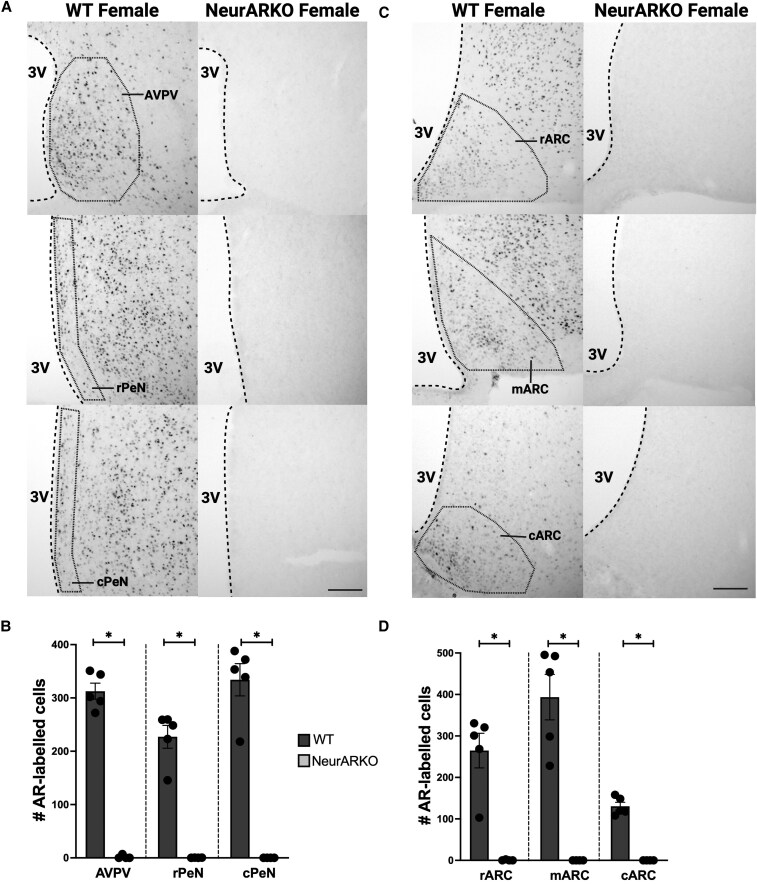
Validation of androgen receptor knock out in hypothalamic neurons in adult female mice. (A) Unilateral representative brightfield images (10× magnification, scale bars = 100 μm) of NiDAB immunohistochemistry labelling AR within the AVPV, rPeN, and cPeN in WT mice and loss of AR-labelled cells in NeurARKO mice. (B) Quantification of AR-labelled cells in the subregions of the RP3V. (C) Unilateral representative brightfield images (10× magnification, scale bars = 100 μm) of NiDAB immunohistochemistry labelling AR within the rARC, mARC, and cARC in WT mice and loss of AR-labelled cells in NeurARKO mice. (D) Quantification of AR-labelled cells in the subregions of the ARC. Data are expressed as mean ± SEM. WT (n = 5) NeurARKO (n = 4), unpaired Student *t*-test, (*P < .0001). Abbreviations: 3V, third ventricle; ARC, arcuate nucleus; AVPV, anteroventral periventricular nucleus; c, caudal; m, middle; PeN, periventricular nucleus, r; rostral.

In WT adult females, AR-labelled cells were detected in the AVPV, rPeN, cPeN, rARC, mARC, and cARC (Fig. 2A and 2C). In contrast, AR-labelled cells were not detected in NeurARKO mice, indicated by a reduction in the number of AR-labelled cells in the AVPV, PeN, and ARC (*P* < .0001, Fig. 2B and 2D), further validating CamKllα-Cre-mediated excision of AR within the hypothalamus.

### Androgen Signaling in Forebrain Neurons is not Required for the Development of PNA-induced Reproductive Features

**Figure 3.**
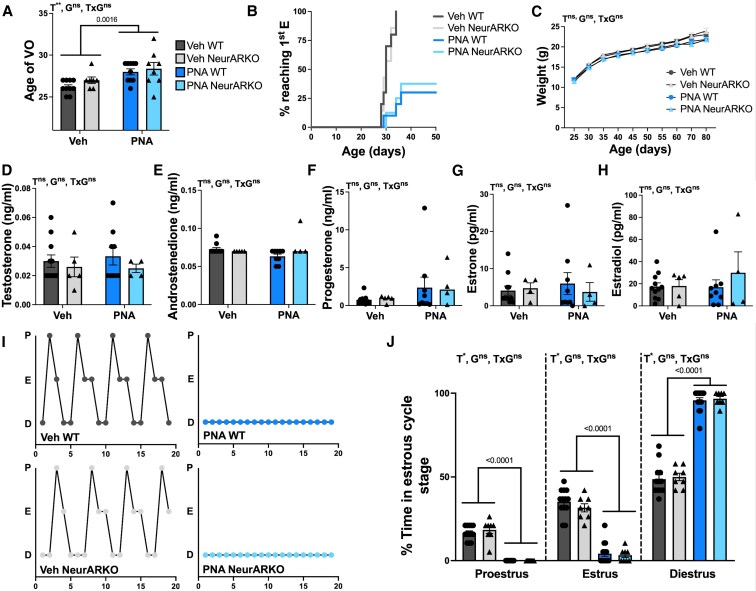
NeurARKO does not affect pubertal timing, testosterone levels, or estrous cycles in vehicle and PNA-treated mice. (A) Age of vaginal opening showing a main effect of PNA (**P = .0016). (B) Cumulative survival plot showing the timing of first estrus for each treatment group. (C) Body weight for each treatment group measured every 5 days from age 25 to 80 years with no main effects. Steroid hormones were measured by LC-MS in each treatment group and comparable serum levels were detected for (D) testosterone, (E) androstenedione, (F) progesterone, (G) estrone, and (H) estradiol. (I) Representative estrous cycle patterns in adult female mice. (J) Percentage of time spent in each stage of the estrous cycle, showing a main effect of PNA (*P < .0001). Analyzed by 2-way ANOVA. Data are expressed as mean ± SEM where circles represent WT and triangles represent NeurARKO. Veh WT (n = 8-13), Veh NeurARKO (n = 5-8), PNA WT (n = 8-15), and PNA NeurARKO (n = 4-8). Abbreviations; D, diestrus; E, estrus; G, NeurARKO genotype; LC-MS, liquid chromatograph-tandem mass-spectrometry; ns, not significant; P, proestrus; T, PNA treatment; VO, vaginal opening.

PNA exposure led to a delay in the age of vaginal opening compared to Veh controls (PNA main effect, *P* = .0016; Fig. 3A). Additionally, although 100% of vehicle-treated mice exhibited first estrus by 34 days of age, less than 50% of PNA-treated mice reached first estrus by 50 days of age (Fig. 3B). There was no effect of PNA or NeurARKO on body weight, measured between postnatal days 25 and 80 (Fig. 3C), indicating that PNA-induced pubertal delay is not attributed to a metabolic influence.

Using LC-MS to measure circulating steroid hormone levels in diestrus, no difference in testosterone, androstenedione, progesterone, estrone, or estradiol were detected between groups (Fig. 3D-3H). All PNA-treated animals were acyclic regardless of genotype, evident by the absence of a proestrus smear and an increase in the percentage of time spent in diestrus compared to vehicle-treated mice (PNA main effect, *P* < .0001, Fig. 3I, 3J).

**Figure 4.**
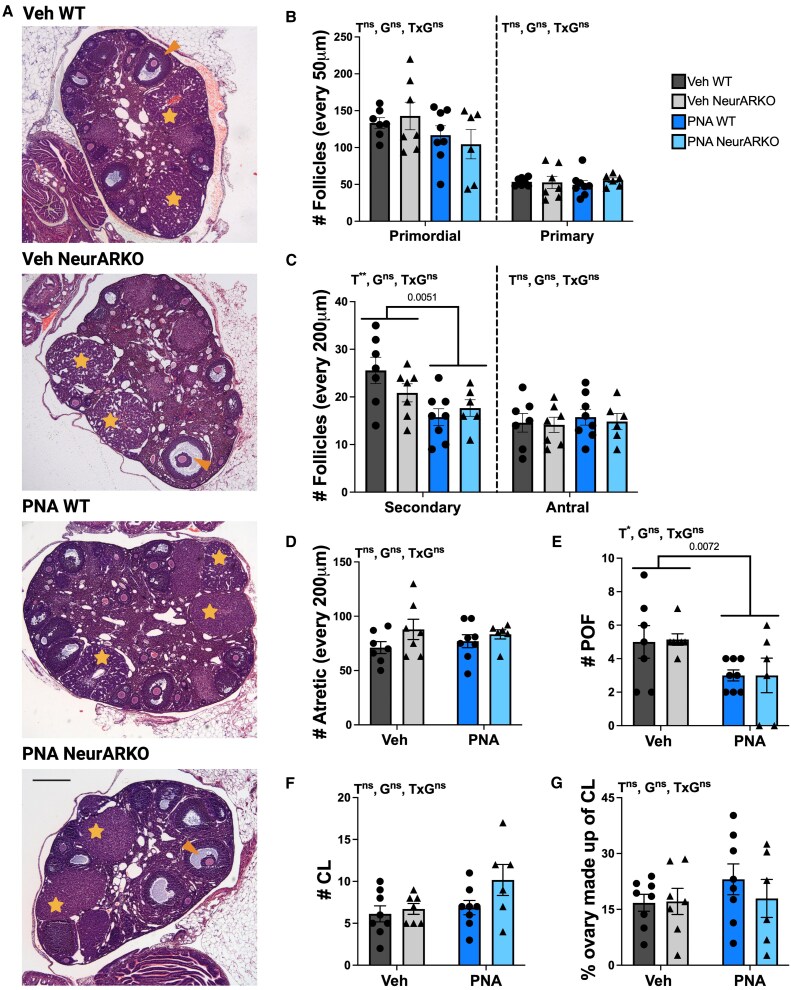
PNA treatment minimally alters ovarian morphology. (A) Representative brightfield images (4× magnification, scale bars = 100 μm) of hematoxylin and eosin-stained ovarian sections from each treatment group. Stars = CL and arrowheads = POF. (B) Number of primordial and primary follicles quantified in sections 50 μm apart in each treatment group with no main effects. (C) Number of secondary and antral follicles quantified in sections 200 μm apart in each treatment group. No main effect of antral follicle numbers and a main effect of PNA on the number of secondary follicles (**P = .0051). (D) Number of atretic follicles quantified in sections 200 μm apart in each treatment group with no main effects. (E) Number of POF quantified in sections 300 μm apart in each treatment group with a main effect of PNA treatment (*P = .0072). (F) The average percentage of the ovary made up of CL tissue in the 2 largest middle sections in each treatment group with no main effects. (G) Number of CL quantified in sections 300 μm apart in each treatment group with no main effects. Analyzed by 2-way ANOVA. Data are expressed as mean ± SEM where circles represent WT and triangles represent NeurARKO. Veh WT (n = 7-8), Veh NeurARKO (n = 7), PNA WT (n = 8), and PNA NeurARKO (n = 6). Abbreviations; CL, corpora lutea; G, NeurARKO genotype; ns; not significant; POF, preovulatory follicles; T, PNA treatment.

Despite the acyclic phenotype of PNA-treated females, minimal modifications of ovarian morphology were detected between groups (Fig. 4A). There was no effect of PNA or NeurARKO on the number of primordial, primary, antral, and atretic follicles (Fig. 4B-4D). However, the number of secondary follicles (PNA main effect, *P* = .0051; Fig. 4C) and preovulatory follicles (PNA main effect, *P* = .0072, Fig. 4E) was reduced in all PNA-treated mice. There was no effect of NeurARKO on these features. In addition, the number of CL and percentage of CL tissue in the 2 largest middle ovarian sections were comparable between groups (Fig. 4F-4G).

### NeurARKO is not Protective for PNA-induced Subfertility

**Figure 5.**
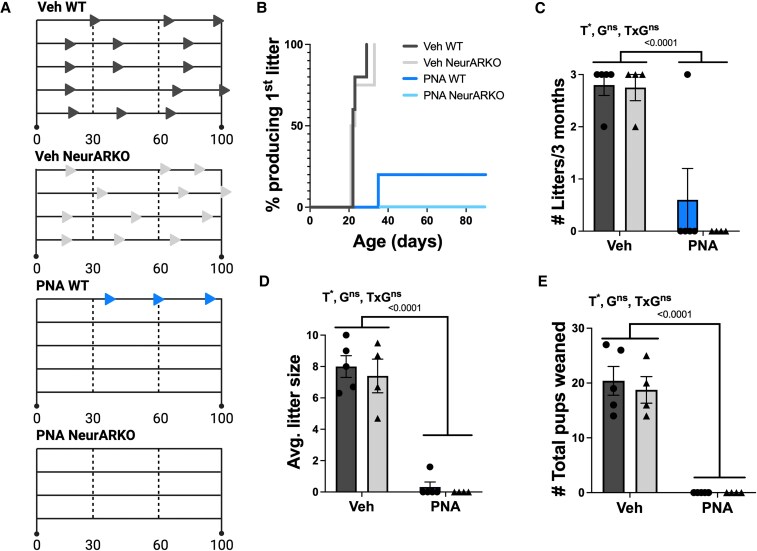
PNA-induced subfertility is not ameliorated in PNA NeurARKO mice. (A) Overview of 3-month breeding trial from each treatment group where each horizontal line represents a mating and triangles represent a litter. (B) Cumulative survival plot showing the percentage and timing of animals to produce their first litter for each treatment group. (C) Number of litters sired over 3 months for each treatment group with a main effect of PNA (*P < .0001). (D) Average litter size at birth across litters produced in 3 months for each treatment group with a main effect of PNA (*P < .0001). (E) Total number of pups that were weaned from litters produced in 3 months for each treatment group with a main effect of PNA (*P < .0001). Analyzed by 2-way ANOVA. Data are expressed as mean ± SEM where circles represent WT and triangles represent NeurARKO. Veh WT (n = 5), Veh NeurARKO (n = 4), PNA WT (n = 5), and PNA NeurARKO (n = 4). Abbreviations; G, NeurARKO genotype; ns, not significant; T, PNA treatment.

A subset of female mice from each experimental group were included in a fertility and fecundity study over a 3-month period (Fig. 5A). All vehicle-treated mice produced litters by day 33, whereas only 1 of 5 PNA WT breeding pairs produced a litter by day 35, and no litters were produced by PNA NeurARKO mice (Fig. 5B). Moreover, all PNA-treated groups produced fewer litters over 3 months (PNA main effect, *P* < .0001, Fig. 5C), a smaller average litter size (PNA main effect, *P* < .0001, Fig. 5D), and a smaller cumulative number of pups weaned (PNA main effect, *P* < .0001, Fig. 5E) compared to vehicle-treated controls.

### Androgen Signaling in Forebrain Neurons is Required for the PNA-induced Downregulation of PR Expression Within the Hypothalamus

**Figure 6.**
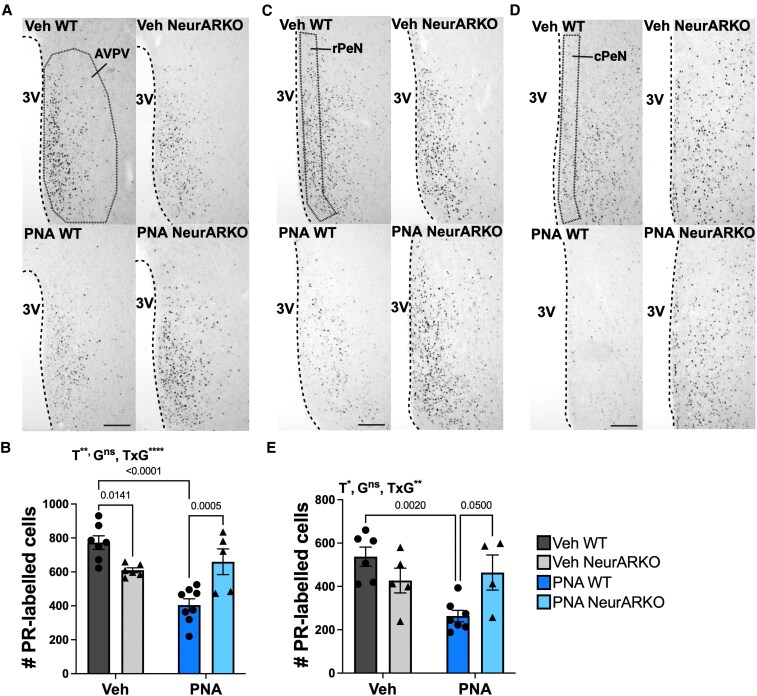
PNA-induced downregulation of PR expression within the RP3V is dependent on androgen signaling in forebrain neurons. Unilateral representative brightfield images (10 × magnification, scale bars = 100 μm) of NiDAB immunohistochemistry labelling PR in sections from the (A) AVPV, (C) rPeN, and (D) cPeN. (B) Quantification of PR-labelled cells in the AVPV for all treatment groups showing a main effect of PNA (**P = .0015) and a PNA × NeurARKO interaction (****P < .0001). (E) Quantification of PR-labelled cells in the PeN from combined counts from the rPeN and cPeN for all treatment groups. Main effect of PNA (*P = .0274) and a PNA × NeurARKO interaction (**P = .0055). Analyzed by 2-way ANOVA and Tukey multiple comparisons post hoc test. Data are expressed as mean ± SEM where circles represent WT and triangles represent NeurARKO. Veh WT (n = 6-7), Veh NeurARKO (n = 5-6), PNA WT (n = 7-8), and PNA NeurARKO (n = 4-5). Abbreviations; 3V, third ventricle; ARC, arcuate nucleus, c, caudal; G, NeurARKO genotype; m, middle; ns, not significant; PeN, periventricular nucleus; r, rostral; T, PNA treatment.

In adult females, NiDAB immunohistochemistry was performed on brain sections to label cells expressing PR within the AVPV, PeN, and ARC, which contain steroid-sensitive excitatory GnRH neurons afferents. In the AVPV, there was an interaction of PNA and NeurARKO (*P* < .0001, Fig. 6A, 6B), with a PNA main effect (*P* = .0015, Fig. 6A, 6B). Post hoc Tukey test revealed that Veh NeurARKO mice had a reduced number of PR-labelled cells compared to Veh WT mice (*P* = .0141, Fig. 6A, 6B). As expected, post hoc Tukey test also revealed that PNA WT mice had a significantly reduced number of PR cells compared to Veh WT (*P* < .0001, Fig. 6B). Interestingly, the number of PR-labelled cells in PNA-treated NeurARKO mice were comparable to vehicle-treated NeurARKO controls (Fig. 6A, 6B). A similar pattern was observed in the PeN which consisted of combined cell counts from the rPeN and cPeN (Fig. 6C-6E). There was an interaction between PNA and NeurARKO (*P* = .0055, Fig. 6C-6E), with a PNA main effect (*P* = .0274, Fig. 6C-6E). The number of PR-labelled cells was decreased in PNA WT mice compared to PNA NeurARKO mice (*P* = .05, Fig. 6C-6E), and comparable to vehicle-treated controls.

**Figure 7.**
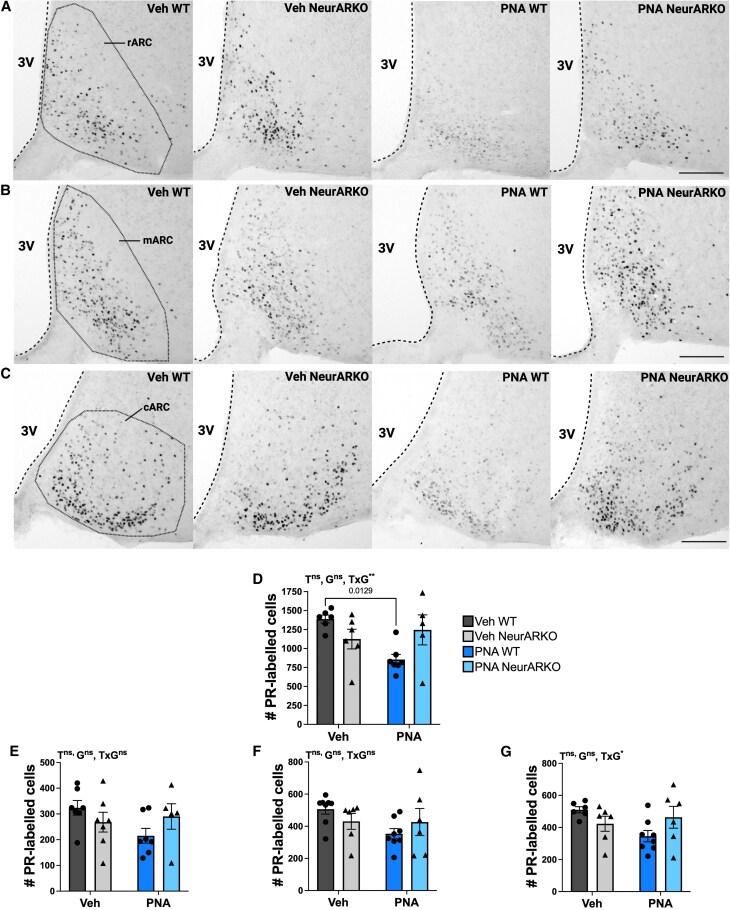
PNA-induced downregulation of PR expression within the ARC is dependent on androgen signaling in forebrain neurons. Unilateral representative brightfield images (10 × magnification, scale bars = 100 μm) of NiDAB immunohistochemistry labelling PR in sections from the (A) rARC, (B) mARC, and (C) cARC. (D) Quantification of PR-labelled cells in the ARC from combined counts from the rARC, mARC, and cARC. PNA × NeurARKO interaction (**P = .0099). (E) Quantification of PR-labelled cells in the rARC for all treatment groups with no main effects. (F) Quantification of PR-labelled cells in the mARC for all treatment groups with no main effects. (G) Quantification of PR-labelled cells in the cARC for all treatment groups with a PNA × NeurARKO interaction (*P = .0332). Analyzed by 2-way ANOVA and Tukey multiple comparisons post hoc test. Data are expressed as mean ± SEM, where circles represent WT and triangles represent NeurARKO. Veh WT (n = 6-8), Veh NeurARKO (n = 6-7), PNA WT (n = 7-8), and PNA NeurARKO (n = 5-6). Abbreviations; 3V, third ventricle; ARC, arcuate nucleus, c, caudal; G, NeurARKO genotype; m, middle; ns, not significant; PeN, periventricular nucleus; r, rostral; T, PNA treatment.

In the ARC, an interaction between PNA and NeurARKO was also identified (*P* = .0099, Fig. 7A-7D). Post hoc Tukey test revealed that the number of PR-labelled cells was reduced in PNA WT mice compared to Veh WT (*P* = .0129, Fig. 7D). The number of PR-labelled cells in PNA NeurARKO mice were not different to PNA WT and vehicle-treated controls (Fig. 7A-7D). To locate the ARC subregion where changes in PR expression occur, the number of PR-labelled cells was assessed in the rARC (Fig. 7A and 7E), mARC (Fig. 7B and 7F), and cARC (Fig. 7C and 7G). There was no main effect of PNA, NeurARKO, or a PNA NeurARKO interaction in the rARC and mARC subregions. In contrast, an interaction between PNA and NeurARKO was detected in the cARC (*P* = .0332, Fig. 7C and 7G). However, post hoc Tukey test revealed no significant pairwise differences. The inability to define the ARC subregion where PR expressional changes are occurring is likely because of the variability in the tissue.

## Discussion

Identifying whether the brain is a primary site of early androgen programming is critical to understanding the developmental origin of HPO axis dysregulation in PCOS. Using a transgenic approach to target AR in forebrain neurons, we found that the NeurARKO genotype was insufficient to ameliorate PNA-induced PCOS-like reproductive features. Delayed pubertal onset, disrupted reproductive cycles, subtle alteration of ovarian morphology, and subfertility were reported in PNA-treated mice, regardless of neuronal AR expression. Collectively, these data indicate that prenatal androgen programming of reproductive dysfunction is not solely mediated by androgen-sensitive forebrain neurons. Contrary to PCOS-like reproductive features, NeurARKO prevented PNA-treated mice from reduced PR expression in steroid-sensitive hypothalamic nuclei, supporting the hypothesis that excessive androgen signaling via neurons directly downregulates PR. Our findings also suggest that prevention of PR downregulation does not alter the severity of PNA-induced reproductive features, further suggesting that impaired sensitivity to progesterone feedback does not solely drive PNA-induced HPO axis dysregulation.

Both clinical and preclinical evidence indicate that PNA is a key etiologic factor associated with programming long-term reproductive dysfunction. The PNA mouse model supports a developmental origin for PCOS, with female offspring exhibiting diagnostic features of PCOS (6, 12, 31). Dysregulation of the HPO axis, evident by elevated GnRH/LH pulsatility and impaired steroid feedback, is also common in PNA mice and women with PCOS (8, 27, 38, 39). Moreover, PNA mice share the highest proportion of differentially regulated genes with PCOS women compared to other murine models, supporting the PNA mouse model as the most translationally relevant to PCOS (40). In the present study, PNA WT mice exhibited well-characterized features of the model including delayed pubertal onset, an acyclic phenotype, and subfertility (8, 11, 12, 25, 31). However, the ovarian morphology of PNA WT mice from our study was minimally altered compared to existing studies reporting reduced numbers of CL and preovulatory follicles, and increased numbers of atretic follicles (11, 31, 41, 42). Sucquart et al (2024) similarly reported no reductions in CL number in AR^fl/fl^ PNA mice. Therefore, there may be off-target effects of the transgenes or an unidentified drift in the model contributing to a subtle influence of PNA on ovarian morphology. Another notable finding was the comparable serum testosterone concentrations between PNA and vehicle-treated mice, potentially indicating model variability and limited statistical power.

Transgenic mice with a neuron-specific deletion of AR, driven by CamKllα-Cre, enabled us to investigate the role of the brain in mediating prenatal androgen excess. AR was successfully excised from hypothalamic neurons in NeurARKO mice, evident by an absence of detectable protein expression in E16 male and female mice, indicating that sufficient CamKllα-Cre expression occurs before E16 to excise AR. Supporting this, CamKllα-Cre expression has been detected at E10.5 in reporter mice, with significant neural expression at E14.5 (43). Hypothalamic AR deletion in NeurARKO mice was also assessed in brain sections from the experimental cohort of adult females where AR protein was undetectable in the RP3V and ARC. Brain-specific AR loss has also been validated by RT-PCR in NeurARKO mice, as previously described (29, 44). Together, our findings indicate that hypothalamic AR deletion has occurred by E16 and is sustained into adulthood. Because the initial exposure to DHT occurs at E16 in the PNA model, we can be confident that neuron-specific AR deletion has occurred in NeurARKO mice.

Despite demonstrating an early and robust loss of forebrain AR in NeurARKO mice, reproductive abnormalities were not detected in vehicle-treated NeurARKO mice, supporting previous indication that AR signaling in forebrain neurons is not required for normal reproductive function (19, 20, 44). In addition, NeurARKO did not protect PNA-treated females from developing PCOS-like reproductive dysfunction, suggesting that the impact of prenatal androgen excess is not solely mediated by androgen-sensitive hypothalamic neurons. This finding is consistent with studies employing AR knockout in specific neuronal populations, where PNA GABARKO mice, and both PPA GABARKO and agouti-related peptide ARKO mice fail to show an amelioration in reproductive features (25, 45). However, the lack of reproductive rescue in PNA NeurARKO mice contrasts previous reports in PPA NeurARKO mice (19). As the number of AR-expressing neurons in the ARC is elevated in PND25 mice compared to E17.5 and PND40 mice (24), the peripubertal period may represent a window of heightened sensitivity to androgens. In addition, NeurARKO may be protective in PPA mice as chronic DHT exposure exerts constant negative feedback that suppresses HPO axis activity, whereas a short window of DHT exposure instead programs impaired sensitivity to steroid hormone feedback in PNA mice.

As prenatal androgen programming of reproductive dysfunction is not exclusively mediated by forebrain neurons, input from the periphery may be a contributing factor. The postnatal deletion of AR from leptin-sensitive cells in neurons and the periphery in PNA mice has been shown to ameliorate acyclicity and ovarian morphology but not delayed pubertal onset (46). Furthermore, interventions targeting metabolic factors such as voluntary running (without weight loss) and treatment with the insulin-sensitizing drug metformin, have been shown to improve reproductive cycling in PNA mice (27, 47). Given that PNA mice exhibit dysfunction across multiple organ systems, PNA may program pathogenic alterations in androgen-sensitive metabolic targets, including leptin-sensitive cells, which then contribute to reproductive dysfunction.

NeurARKO, although not effective in limiting the development of PCOS-like reproductive traits, prevented the PNA-induced downregulation of hypothalamic PR expression. These data raise several points of discussion. First, this blockade of a known PNA outcome supports the hypothesis that excessive androgen signaling in GnRH neuron afferents directly inhibits PR expression, and likely the sensitivity of these neurons to progesterone feedback (8, 23). This mechanism may involve nuclear AR-mediated inhibition of PR gene transcription, consistent with evidence showing reduced PR mRNA detection in the brain following exogenous testosterone administration (48). Second, as rescue of hypothalamic PR expression without an amelioration of reproductive features was identified in PNA NeurARKO mice, impaired sensitivity to progesterone feedback in the hypothalamus alone is unlikely to underpin HPO axis dysregulation associated with PCOS. It is also important to consider whether the observed partial loss of PR in PNA WT mice is significantly contributing to the observed neuroendocrine phenotype, and therefore, whether the restoration of PR expression in PNA NeurARKO may not have a significant impact.

Together, our findings suggest that forebrain neurons may not solely mediate PNA programming of PCOS-like reproductive features. However, because AR deletion occurred during embryonic development, compensatory mechanisms cannot be ruled out. In addition, this study supports a role for neuronal AR in directly programming downregulation of PR within the hypothalamus. As both PNA mice and hyperandrogenic females have reported reduced sensitivity to progesterone feedback (4, 31, 49, 50), our findings suggest that impaired steroid hormone feedback may be a consequence, rather than driving factor of PCOS-like reproductive dysfunction. Given that women with PCOS often present with cardiometabolic, immune, and psychological features in addition to reproductive dysfunction, targeting the brain alone may not account for the full rescue of PCOS. Therefore, identifying tissue-specific effects of androgen excess directly following PNA programming and in peripheral tissues is critical for further understanding the mechanism of HPO-axis dysfunction associated with PCOS.

## Data Availability

Some or all datasets generated during and/or analyzed during the current study are not publicly available but are available from the corresponding author on reasonable request.

## Abbreviations

AR: androgen receptor
ARC: arcuate nucleus
AR^fl/fl^: AR-floxed mice
ARKO: AR knockout mice
AVPV: anteroventral periventricular nucleus
cARC: caudal arcuate nucleus
CL: corpora lutea
cPeN: caudal periventricular nucleus
DAB: 3,3′-diaminobenzidine
DL: detection limit
E: embryonic day
HPO: hypothalamic-pituitary-ovarian
LC-MS: liquid chromatography-tandem mass-spectrometry
mARC: middle arcuate nucleus
NeurARKO: neuron-specific knockout of androgen receptor
PCOS: polycystic ovary syndrome
PFA: paraformaldehyde
PNA: prenatal androgen excess
PND: postnatal day
PPA: peripubertally androgenized
PR: progesterone receptor
PVN: paraventricular nucleus
PVT: paraventricular nucleus of the thalamus
rARC: rostral arcuate nucleus
rPeN: rostral periventricular nucleus
Veh: vehicle
WT: wild-type
